# Scarification of the Gums during Dentition

**Published:** 1844-10

**Authors:** Marshall Hall


### PRACTICAL MEDICINE, &c.

*Scarification of the Gums during Dentition.* By Marshall Hall, M.D., F.R.S., &c.—There is no practical fact of the truth and value of which I am more satisfied than that of the effect and efficacy of scarification of the gums in infants, and not in infants only, but in children. But the prevailing, I may say the univer-

*Cooper's Surg'l Dict.; Art. Prostate Gland.

sal idea on the subject is, that we should lance the gums only when the teeth are ready to pierce through them, and only at the most prominent parts of the gums, and as the occasion to which I have referred may require; and no idea of this important measure can be more inadequate to its real value. The process of teething is one of augmented arterial action and of vascular action generally; but it is also one of augmented nervous action; for formation, like nutrition, secretion, &c., generally, is always one of nervi-vascular action, and of this the case in question is, from its peculiar rapidity, one of the most energetic. Like other physiological processes, it is apt to become, from that very character of energy, pathological, or of morbid activity. It is obviously, then, attended with extreme suffering to the little patient; the brain is irritable, and the child is restless and cross; the gums are tumid and heated; there is fever, an affection of the general vascular system, and there are, too frequently, convulsions of various degrees and kinds, manifested in the muscles which move the eyeball, the thumb and finger, the toes; the larynx, the parietes of the respiratory cavities; and the limbs and frame in general; affections of the excitomotor part of the nervous system, and of the secretions of the liver, kidneys, and intestines; affections of the ganglionic division of that system.

What is the precise cause and source of these formidable effects? Can the mere tension and irritation of the gum situated over the more prominent part of the teeth be the cause of such extensive morbid actions? I think not. The real source of these phenomena is in the entire dental system, in which actions of unusual energy and extent are going on—sub-inflammatory they might be called, were they not in reality of an essentially different nature and origin. This undue action takes place in the fangs and sockets of the teeth in their whole extent, with their connections, vascular, nervous, and membranous. But *the* focus from which the *nervous* actions emanate is, I believe, not as is generally imagined, the nerves of the mere *gums* seated over the piominent parts of the teeth, but the nerves which may emphatically be termed *the nerves of the teeth themselves*, the nerves which enter into the very fangs and substance of the teeth. *It is to the base of the gums, not to their apex merely, that the scarification should be applied.* The most remarked case in which I have observed the instant good effect of scarification was one in which *all the teeth had pierced the gums!*

This view of the subject may assist in removing the futile objection of some who have, without due consideration I am convinced, opposed my plan of frequent, often daily, scarification of the gums, to whom I would say, as my sole reply—Better scarify the gums *unnecessarily* one hundred times, than allow the accession of one fit or convulsion from the neglect of this operation, which is equally important in its results, and trifling in its character. And it is not merely the prominent and tense gum over the edges of the teeth which should be divided; the gums, or rather the blood-vessels, immediately over the very *nerves of the teeth*, should be scarified and divided, as you would divide the vessels of the conjunctiva in inflammation of that membrane.

Now, whilst there is fever or restlessness, or tendency to spasm or convulsion, this *local blood-letting* should be repeated daily, and and in urgent cases even twice a day. I would here repeat my maxim—Better do this one hundred times unnecessarily than have one single fit from the neglect of so trifling an operation. A skilful person does it in minute, and in a minute often prevents a most serious attack—an attack which may cripple the mind or limbs, or even take the life of our little patient, if frequently repeated. There is, in fact, no comparison between the means and the end, the one so trifling, the other so momentous.

I would refer those who wish to prosecute this subject to my work on the "Diseases and Derangements of the Nervous System," but especially to my "New Memoir," which contains the most lucid and recent view of the whole subject of the physiology and pathology of the true spinal system, and plates which, for skill in the draughtsman (Mr. Simpson, of Stamford) both that of the artist and that of the physician, and for interest in a practical point of view, have not been surpassed. *Each* plate evolves a principle of physiology or pathology of great interest and value.

I have frequently thought the vascular condition of the gums during dentition might be ascertained by means of a thermometer properly guarded. The results of a series of observations on this point could not fail to possess much value, whilst they would probably suggest a means of diagnosis in some serious disease. I do not pretend, in the above proposition, to have advanced anything new; but in the *locality* chosen for the operation, and in the *promptitude, repetition, perseverance*, and in the *energy and steadiness of purpose* with which I recommend the measure to be adopted—if these be fully apprehended—I believe I do propose something *new;* and when I repeat that since I adopted the plan of *effectually* removing *all* irritation in the gums, stomach, and intestines, in cases of crowing and other convulsions of the same nature, early enough, I have not known or seen a fatal case, I am aware that I propose a plan of treatment at once new and *valuable.* But half measures are of no efficacy. These remarks do not apply, of course, to convulsive diseases of centric origin.—*Braithwaite's Med. Retrospect, from Lancet, May* 8, *p.* 414. 1844.

